# Adenomyosis: Disease, uterine aging process leading to symptoms, or both?

**Published:** 2020-08-05

**Authors:** A Protopapas, G Grimbizis, S Athanasiou, D Loutradis

**Affiliations:** 1st Department of Obstetrics & Gynecology of the Medical School of the National and Kapodistrian University of Athens, Greece;; 1st Department of Obstetrics & Gynecology of the Medical School of the Aristotle University of Thessaloniki, Greece.

**Keywords:** Adenomyosis, diagnosis, epidemiology, pathogenesis symptoms

## Abstract

For many decades adenomyosis has been a histological diagnosis in hysterectomy specimens. Traditionally, it has been considered a disease of late reproductive and premenopausal years causing uterine enlargement, dysmenorrhoea and menorrhagia. Recent advances in pelvic and uterine imaging techniques including transvaginal sonography and magnetic resonance imaging were responsible for a shift towards a non-invasive diagnosis and made a significant contribution to a better understanding of its pathogenesis, epidemiology, histological spectrum, and clinical symptomatology. With these non-invasive tools it has been shown that adenomyosis is probably a condition affecting much younger populations and is frequently asymptomatic at an early stage of its development. Regarding symptomatic disease, the distribution and extent of adenomyotic lesions do not correlate consistently with the various symptoms that are considered typical of adenomyosis. More importantly, accurate diagnosis of adenomyosis suffers from a lack of consensus among experts on imaging and even histological diagnostic criteria. Several pathogenetic theories have attempted to shed light on the establishment, evolution and distribution of adenomyotic lesions within the uterine wall, including the tissue injury and repair (TIAR) mechanism, metaplasia, and the more recent genetic-epigenetic theory. So far, none of these can adequately and independently explain the appearance of all types of adenomyosis. This review paper attempts a correlation between the proposed pathogenetic theories and the clinical and histological spectrum of adenomyosis, in an effort to give a plausible explanation of the evolution of this condition from an asymptomatic state to a disease, through synthesis of the existing data.

## Introduction

Adenomyosis is a uterine condition that is histologically characterized by the presence of ectopic endometrial glands and stroma within the myometrium, surrounded by hypertrophic and hyperplastic myometrial changes (Garcia and Isaackson, 2011). For several decades, the diagnosis of adenomyosis was made in hysterectomy specimens either coincidentally, or in women treated surgically for chronic pelvic pain and/or abnormal uterine bleeding (Molitor, 1971). Over the past twenty years more and more cases of adenomyosis are diagnosed with non-invasive methods such as transvaginal 2- or 3-dimensional sonography (2-D TVS and 3-D TVS), or magnetic resonance imaging (MRI) (Andres et al., 2018; Tellum et al., 2020).

These imaging methods have been pivotal in clarifying the functional anatomy of the uterus, changing our understanding of the natural history and the clinical spectrum of adenomyosis significantly. The myometrium is composed of two separate layers; the inner myometrium or junctional zone (JZ), and the outer myometrium, that are histologically and embryologically different (Brosens et al., 1995; Fusi et al., 2006). The inner myometrium, like the endometrium, is of Müllerian origin, undergoes cyclical changes in response to hormonal stimuli, and is involved in embryo implantation and placentation (Uduwela et al., 2000). This area according to the tissue injury and repair (TIAR) pathogenetic theory represents the original site of the development of the adenomyotic process (Leyendecker et al., 2009). Other investigators have suggested that the so- called endometrial-subendometrial unit disruption disease should be considered a separate entity from adenomyosis (Tocci, et al., 2008).

The histopathological spectrum of adenomyosis includes diffuse and circumscribed lesions that may have a variable distribution and extent within the myometrium. This histological variability probably relates to the variety of clinical manifestations that have been attributed to adenomyosis, including the absence of symptoms in many patients (Peric and Frazer, 2006). The time of the 1st appearance and the age-related evolution of adenomyotic lesions is a matter of controversy. A life-cycle approach to both endometriosis and adenomyosis has indicated that these two conditions, despite their common features, have a different epidemiology (Benagiano et al., 2015). Adenomyosis until recently was considered a disease of older women. Nevertheless, advances in imaging techniques have enabled the detection of subtle adenomyotic lesions in very young asymptomatic populations, and this has raised reasonable doubts on whether adenomyosis - or at least some of its forms - is a true disease, or a normal process related to, and aggravated by, uterine aging.

The purpose of this paper is to review the existing data correlating the clinical presentation with the histological and imaging features of adenomyosis, to examine how symptoms may evolve with age, and to attempt a correlation of clinical manifestations of adenomyosis with existing theories of pathogenetic mechanisms. Using a systematic approach, we formulated a hypothesis that considers adenomyosis to be a multi-faceted entity which, in accordance with all principal pathogenetic theories, may be diagnosed throughout a woman’s life, acquiring the characteristics of a morbid condition, when significant molecular changes occur and symptoms develop. This transformation may or may not be age-related and will depend on the type of lesion, the mechanism of its initial development in an ectopic location, and the sustained action of important risk factors contributing to its evolution and spread.

## Materials and Methods

A comprehensive search was performed on PubMed, EMBASE, Web of science, and Science Direct for studies reporting on adenomyosis (Title) AND (symptoms OR presentation OR clinical spectrum), adenomyosis symptoms (Title) AND pathogenesis, adenomyosis symptoms (Title) AND epidemiology and adenomyosis symptoms (Title) AND diagnosis, till December 2019. The title and abstract were screened and the full text of 245 possibly relevant articles were assessed by two authors (AP and SA). A total of 89 articles were finally included in this review. The main inclusion criterion was relevance to the question posed in the title of our manuscript. Studies reporting on symptoms of adenomyosis represented the core literature that had been initially built and carefully reviewed. Subsequently, papers correlating symptoms with epidemiology, pathogenesis, and diagnosis of adenomyosis both histological and imaging were reviewed and cross-referenced. There were no particular exclusion criteria. Nevertheless, papers reporting purely on imaging diagnosis without reference to symptoms, were included only after reading the abstract and/or text. Papers on surgical methods to treat adenomyosis were considered only if they included data on pre- and postoperative symptoms. A hypothesis has also been formulated on the evolution of symptoms attributed to adenomyosis, from menarche to menopause, correlating it with existing pathogenetic theories.

## Results

### Pathogenesis and distribution of lesions

In common with endometriosis, it is difficult to cover all cases of adenomyosis under the same pathogenetic umbrella. The TIAR theory suggests that both conditions are the result of trauma which is induced by chronic uterine peristaltic activity or phases of hyperperistalsis at the endometrial- myometrial interface activating a mechanism of tissue injury and repair (Leyendecker et al., 2009). This in the case of adenomyosis is followed by invasion of the endometrium into the myometrium and development of chronic inflammation. Despite being an attractive theory, TIAR may explain many but not all ectopic lesions. Variable depth of lesions in particular may indicate either the operation of different pathogenetic mechanisms, or different stages of the disease process (Leyendecker et al., 2015). Invasion of the breached junctional zone by hyperplastic endometrium and sustained hyper- peristaltic activity, at least initially, would result in superficially located lesions (Garcia-Solares et al., 2018). There is no solid proof that the same process can result in adenomyotic lesions up to the distant outer myometrium. Probably, the theory of de novo development of adenomyotic lesions from metaplasia either as a result of Müllerian remnants, or from external invasion of progenitor epithelial and stromal cells derived from endometrial menstrual debris is more appropriate to explain lesions far distant from basal endometrium and close to the uterine serosa (Garcia-Solares et al., 2018; Gargett, 2016). Kishi Y et al., have suggested an MRI-based classification of adenomyosis into four subtypes, according to involvement or not of the inner and outer layers of myometrium, and separating cases with lesions occurring alone unrelated to structural components, and those not satisfying the above criteria (Kishi et al., 2012). Similarly, Bazot M, and Darai E, classified lesions into internal and external adenomyosis, and structural-related adenomyoma subtypes, but underlined that all three types can be present alone or in association in the same patient (Bazot and Darai, 2018). These two proposed classifications indicate the operation of at least two different pathogenetic mechanisms that may act independently of each other, but at times together on the same subject, giving rise to the complex histological profile of adenomyosis. Co- existence of endometriosis may act as the bridging factor between internal and external adenomyosis, with the TIAR mechanism acting to promote simultaneous appearance of both conditions, during the initial stages of their development (Leyendecker et al., 2009). At a more advanced stage external infiltration of the myometrium by endometriotic stem cells and metaplasia especially in cases with deep endometriosis, may result in the active distant myometrial lesions that constitute the typical adenomyotic foci of the outer myometrium.

The location of ectopic lesions within the different layers of the myometrium no matter how they end up there, may have significant implications on the appearance of symptoms, their quality, and their timing along the natural history of adenomyosis (Bird et al., 1972; Levgur M, et al., 2000; Sammour et al., 2002; Li et al., 2014). The recent genetic-epigenetic theory initially proposed to explain pathogenesis of the different forms of endometriosis (Koninckx et al., 2019), can be equally applied to adenomyosis, as these two conditions share many molecular, immunological and biochemical alterations of the eutopic and ectopic endometrium (Benangiano and Brosens, 2011; Vannuccini et al., 2017). According to this theory, ectopic adenomyotic lesions of variable origin, bearing genetic and epigenetic stigmata, will become a disease after exposure to a toxic environment that will inflict further crucial hits and molecular changes.

To appropriately study the clinical course and imaging evolution of adenomyosis in the aging female we would possibly need to follow-up closely a large cohort of young asymptomatic women from even before menarche to their early postmenopausal years. Such a study does not exist so far, and it would be difficult if not impossible to conduct in the future. In contrast to what is really necessary to further elucidate the pathogenesis of adenomyosis, our assumptions are actually based on studies that currently offer captures of the adenomyotic process, in women of different age groups.

### Diagnosis of adenomyosis: an urgent need for solid criteria

#### 

Traditionally, the diagnosis of adenomyosis had been a histological one made at hysterectomy specimens. At present time, the evolution of imaging tools and especially ultrasound and MRI, has permitted accurate non-invasive diagnosis, using well described morphological myometrial alterations, measurement of the thickness, and assessment of the outline of the JZ, or a combination of all these parameters (Bazot and Darai, 2018, Tellum et al., 2020). Nevertheless, diagnostic criteria, both histological and imaging, have been variable in the existing literature, and this variability could explain to a certain extent significant differences in the observed prevalence of this condition, in groups of patients with similar epidemiological and clinical characteristics.

#### A. Histological diagnosis

Unfortunately, even today, no universally acceptable histological criteria exist. The histological diagnosis commonly relies on the minimum distance from the endometrial-myometrial junction that ectopic lesions are found within the myometrium, but this varies from 1-3mm in reported series. Accordingly, a low or a high-power field (LPF-HPF) has been used as marking reference of depth (Garcia and Isaackson, 2011; Benagiano et al., 2015). This lack of solid histological criteria would significantly affect the reported prevalence and incidence of adenomyosis in different patient populations. Equally, it would affect any subsequent clinical correlations.

In their important study Bird et al. (1972) proposed a histological classification of adenomyosis based on the depth of myometrial invasion and the number of ectopic lesions within the myometrium. In a series of 200 hysterectomies, they showed that the incidence of adenomyosis would increase from 31% to 38.5% if they used 6 extra sections to their routine histological assessment, and more importantly, by including sub-basal lesions (Grade I disease, or adenomyosis sub-basalis, according to their definition), the incidence would rise to an impressive 61.5% (Bird et al., 1972).

Similarly, in a more recent study, Bergholt et al. (2001) reporting on 486 hysterectomised patients, found that by increasing the depth of myometrial invasion from 1mm to 3mm, and including myometrial hyperplasia as essential criteria for the diagnosis of adenomyosis, its prevalence in their cohort would drop from 18% to 10%. Therefore, it is clear that the application of stricter histological criteria for the diagnosis of adenomyosis would significantly affect its reported epidemiology.

#### B. Imaging diagnosis

The development of high-resolution imaging techniques has profoundly affected both our understanding of adenomyosis and the frequency of its diagnosis. But it has also created more problems that need to be addressed. Criteria for the diagnosis of this condition have been established by several groups for all 3 modalities; two and three dimensional transvaginal ultrasonography (2D-TVS and 3D- TVS), and magnetic resonance imaging (MRI) (Reinhold et al., 1996; Bazot et al., 2001; Dueholm, 2006; Exacoustos et al., 2011; Stamatopoulos et al., 2012; Tellum et al., 2019). It is beyond the scope of this article to perform a systematic review of existing studies and discuss the reliability of their diagnostic criteria. On average, they have a good reported sensitivity of 70-80%, and an even better specificity of 80-90% (Champaneria et al., 2010).

In evaluating their performance in the diagnosis of adenomyosis, these modalities have initially been compared with a histological diagnosis made at hysterectomy, which is considered the gold standard. This has three weaknesses: a) the number of women finally submitted to hysterectomy usually represents a minority of the total cohort, b) the number of the imaging criteria considered essential for establishment of a non-invasive diagnosis may vary significantly between studies, and c) the histological criteria as explained above are also subject to variation.

An additional drawback of non-invasive diagnosis is that the population of women subjected to an imaging study and the indication for it. Naftalin et al. (2012) studied a cohort of 986 women visiting a general gynaecology clinic with a variety of complaints with 2D and 3D TVS. They applied seven ultrasonographic criteria for a diagnosis and found a prevalence of 21% of adenomyosis in their population. Only 45 women were finally subjected to a hysterectomy and of these 18 (40%) had co-morbidities such as uterine malignancies or multiple fibroids that complicated assessment of the specimen and were excluded from comparison between ultrasound and hysterectomy.

Another important prospective study published by an experienced Italian Group produced very interesting results reporting on a much different population. Using very strict exclusion criteria Pinzauti et al. (2015) applied 2D and 3D TVS on 156 young women (18-30 years old) attending a contraception clinic. Their ultrasonographic criteria had been previously tested and evaluated. Surprisingly, they found a prevalence of diffuse adenomyosis of 33% (53/156) in a group of women not seeking advice for symptoms. Understandably, no hysterectomies were performed in this cohort of patients.

The Morphological Uterus Sonographic Assessment (MUSA) group, have recently reported on the sonographic features and use of terminology for describing the two most common myometrial lesions (fibroids and adenomyosis) and uterine smooth muscle tumours. Regarding adenomyosis, they have concluded that this condition may be difficult to diagnose with ultrasound. Although different ultrasound features have been suggested to be associated with adenomyosis, at present, it is not clear which of the various ultrasound criteria are most important for diagnosis. Some features may carry a greater diagnostic weight than others and the presence of more than one ultrasound feature associated with adenomyosis might increase the likelihood of the diagnosis. They did not include in their consensus statement the so called ‘question-mark sign’, suggested to be typical of adenomyosis, because this sign occurs when there is also deep infiltrating endometriosis in the posterior compartment. (Van den Bosch et al., 2015).

MRI has been shown to be equally effective – if not better – compared with ultrasound in the diagnosis of adenomyosis, (Bazot and Darai, 2018; Tellum et al., 2020), but it is an expensive tool, and its routine use cannot be justified especially in asymptomatic populations. Nevertheless, the prevalence of adenomyosis in asymptomatic women has been examined using MRI criteria in two studies. In the first study, Hauth et al. (2007) performed MRI in 100 women and found adenomyosis in 12%, whereas Juang et al. (2007) reported on the incidence of adenomyosis postpartum in women with term and preterm deliveries and found an incidence in these two populations of 9.4% and 13.2%, respectively.

In symptomatic women in whom MRI could be much more easily justified, the prevalence of adenomyosis appears significantly different. Four large prospective studies have compared MRI performance with histopathology for the diagnosis of adenomyosis (Reinhold et al., 1996; Bazot et al., 2001; Dueholm et al., 2001; Tellum et al., 2019). These studies give a sensitivity of between 70% to 93% and a specificity of 86 to 93%, with a prevalence of adenomyosis of 21 to 33%. Nevertheless, not all these reports agreed in regarding the usefulness of different diagnostic criteria (thickness and appearance of JZ, and morphological alterations of myometrium). JZ thickness ≥12mm, a finding commonly used to diagnose adenomyosis has been disputed recently by Tellum et al. (2019) who reported that presence of JZ irregularity rather than thickness, and specific morphological criteria such as cysts and adenomyomas provide the highest specificity for diagnosing adenomyosis.

In their meta-analysis comparing the diagnostic performance of MRI and TVS, Champaneria et al. (2010) reported that MRI had a pooled sensitivity of 77% (95% confidence interval (CI) 67–85), a specificity of 89% (95% CI 84–92), a positive likelihood ratio of 6.5 (95% CI 4.5–9.3), and a negative likelihood ratio of 0.2 (95% CI 0.1–0.4). The authors concluded that MRI performs more favourably than TVS in the presence of associated uterine leiomyomas. However, while MRI is less operator-dependent than TVS, expertise is required. Little data are available on the value of MRI to determine the location, severity and extent of adenomyosis in comparison with histology (Reinhold et al., 1996; Dueholm et al., 2001; Rasmussen et al., 2019).

The above data underline the difficulty in attempting to make clinical correlations when no solid criteria for the diagnosis of adenomyosis have been agreed upon among pathologists and imaging experts. Furthermore, the type of population under study, the presence or absence of symptoms attributed to adenomyosis, and any co-existing gynaecological pathology that may complicate the clinical picture and imaging diagnosis, would affect significantly the true prevalence of this disease. An important point regarding non-invasive diagnosis of adenomyosis is how many criteria should be present in each patient to make a certain diagnosis (Naftalin et al., 2012). By using a single imaging feature, women in the normal spectrum of uterine anatomy will be included, whereas by increasing the number of essential criteria, some women with the disease will escape diagnosis (Kepkep et al., 2007). Another important parameter is how to handle the information and how to transfer it especially to young asymptomatic patients or those with minimal symptoms, not requiring therapeutic interventions.

### Clinical symptoms of adenomyosis and correlation to histology and imaging

The reported clinical presentation of adenomyosis is also variable, with severity of particular symptoms not always corresponding to the extent and severity of the disease. Bird et al. (1972) attempted a correlation of the distribution and number of adenomyotic lesions within the myometrium with the different symptoms reported by their patients. They found that menorrhagia was more common in patients with Grade I disease in comparison with those with deeper disease affecting the middle and more distant myometrium – Grades 2 and 3 disease (60% vs. 42%, respectively). The severity of involvement, indicated by the number of glands per LPF, was significantly associated with the frequency of menorrhagia. When >10 glands/LPF were found, menorrhagia was present in 82% of cases, compared to 58% (4-9 glands/LPF), and 23% (1-3 glands/LPF), of a lesser myometrial involvement. On the contrary, the rates of severe dysmenorrhea increased proportionally, according to the depth of myometrial involvement (4.3% vs. 42.4% vs. 83.3%). Increasing disease severity defined by the number of ectopic lesions within the myometrium also significantly affected the rates of dysmenorrhea (13.3% vs. 26.7% vs. 58.8%). Nevertheless, only 18.7% of their patients had both menorrhagia and severe dysmenorrhea – the classic symptom complex. Their findings underscore the potential significance of lesion depth and location (inner vs. outer myometrium), on the type and severity of symptoms accompanying the presence of adenomyosis, and possibly the timing of their appearance during the evolution of the adenomyotic process.

In a more recent study, Levgur et al. (2000) reported on 111 uteri weighing <280gr that were assessed with full-thickness sections, of these, 36 had adenomyosis. According to the depth ectopic lesions were found in relation to total myometrial thickness, they graded adenomyosis as superficial (<40%), intermediate (40-80%), and deep (>80%). The median number of foci was higher in women with dysmenorrhea compared with those without the symptom. On the contrary, there was no difference in the number of foci in women with and without menorrhagia. Superficial adenomyosis was not associated with either menorrhagia or dysmenorrhea. Their findings partly contradict those of Bird et al. (1972) but it should be mentioned that they did not include cases with <2.5mm superficial myometrial involvement in their analysis, nor cases with uteri weighing >280gr, for technical reasons, i.e. difficulty to achieve full-thickness sections.

Sammour et al. (2002) also reported on 92 cases with adenomyosis diagnosed at hysterectomy. They classified myometrial involvement by adenomyosis into 4 groups, each corresponding to 25% of myometrial thickness, excluding superficial foci <2mm from the basal endometrium. They reported a lack of correlation between symptoms and the depth of adenomyosis, whereas they found a significant correlation of pelvic pain or dysmenorrhea, but not menorrhagia and dyspareunia, with the spread of the adenomyotic lesions.

In a large Chinese study, which included 770 cases of adenomyosis diagnosed at hysterectomy out of a total of 1690 patients, Li et al. (2014) reported on the correlation of symptoms attributed to adenomyosis with the age of 1st appearance, and their severity. They found that dysmenorrhea was the most common symptom reported by 81.7% of patients either alone or in combination with other complaints. Severe dysmenorrhea in particular, was found to be significantly associated with a younger age of 1st diagnosis of adenomyosis, appearance of symptoms at a lower age, its presence as a sole symptom, and with a longer duration of symptoms. Menorrhagia appeared later in life affecting women in their mid-40s and was commonly associated with other symptoms, and in particular severe dysmenorrhea. Asymptomatic women with adenomyosis (only 4.5% in this series) were predominantly of the late premenopausal age range. In this study the size of the uterus did not differ significantly between symptomatic and asymptomatic women, in agreement with the findings of a previous study by Molitor et al. (1971). In contrast, Bird et al. (1972) had found that adenomyotic uteri were on average heavier than normal.

The above correlations should be viewed with caution for the simple reason that patients with adenomyosis submitted to a hysterectomy are commonly of the older age group and complain of more severe symptoms, have more co-morbidities causing similar symptoms, and probably are not representative of the true clinical spectrum of this disease. Furthermore, differences in methodology such as criteria for the histological diagnosis of adenomyosis and number of sections used, indications and threshold for hysterectomy, and the impact of the healthcare system may have significantly affected the above correlations.

It is clear that patients with adenomyotic changes diagnosed with imaging methods may tell a different story regarding adenomyosis-related symptoms. Naftalin et al. published two subsequent studies on practically the same patient population of a general gynaecology clinic (mean age: 38 years, inter-quantile range - IQR: 30-43), in an attempt to correlate TVS findings suggestive of adenomyosis with both dysmenorrhea and menorrhagia (Naftalin et al., 2014; Naftalin et al., 2016). They found that the increasing number of a panel of seven ultrasonographic criteria present in each case was significantly associated with a worsening dysmenorrhea and with the severity of menorrhagia. Nevertheless, not all ultrasonographic characteristics had the same importance regarding the severity of symptoms, nor did it become clear how many should constitute a certain diagnosis of adenomyosis, taking into account the wide variability of their presence in patients with a diagnosis of this disease (from 1.3-26.8%). Kepkep et al. (2007) in their study of 70 patients correlating ultrasonographic and histological diagnosis of adenomyosis, found that imaging characteristics have variable sensitivities, specificities, and negative and positive predictive values.

In agreement with the above findings, Pinzauti et al. (2015) in their study on much younger nulligravid patients (mean age: 24 years, IQR: 23- 27) attending a contraception clinic found that the number of ultra-sonographic findings suggestive of adenomyosis at 2D-TVS, and the thickness of the JZ on a coronal section at 3D-TVS, both correlated significantly with the severity of dysmenorrhea and menorrhagia assessed by visual analogue scale (VAS) and pictorial blood loss analysis chart (PBAC) scores, respectively. Nevertheless, the mere diagnosis of adenomyosis using the presence of a single ultrasonographic feature was not associated with the subjective symptom of menorrhagia. Although the obvious weakness of this study is the lack of histological confirmation of the diagnosis of adenomyosis, finding evidence of diffuse adenomyosis in a significant proportion (1:3) of young nulligravid women without obvious classic risk factors (previous pregnancy and labor, miscarriage, uterine surgery, IUCD use), casts doubt on the true pathogenetic pathways leading to development of this disease.

Recently, Exacoustos et al. (2019) reported on 108 patients with ultrasonographic signs of adenomyosis (mean age 37.7±7.7 years) who were classified according to a proposed scoring system that graded the type of adenomyosis (diffuse vs. focal) and its extension inside the myometrial wall. Women with ultrasound diagnosis of diffuse adenomyosis were older (p= 0.04) and had heavier menstrual bleeding (p=0.04) than women with focal disease, however no statistically significant differences were found regarding the presence and severity of dyspareunia and dysmenorrhea. Higher values of menstrual bleeding were found for severe diffuse adenomyosis and the highest values were found in those with adenomyomas.

An important relevant issue is whether ultrasonography can correctly identify the grade or degree of adenomyosis. Bazot et al. (2002) in an older study, reported concurrence between histopathology and TVS in only 57% of cases, when assessing the depth of presence of endometrium within the myometrium, and in only 23% of cases, when assessing the degree of involvement and lesion density. This relative weakness of ultrasonography would probably negatively affect any effort to classify adenomyosis and its severity with imaging, and subsequently any clinical correlations, made important by previous studies reporting on histopathological diagnosis (Bird et al., 1972; Bergholt et al., 2001). Unfortunately, few recent studies using modern imaging (ultrasound and MRI) equipment have attempted correlations between detailed imaging and extensive histological sections in large hysterectomy populations. Rasmussen et al. (2019) have recently reported on 110 patients submitted either to hysterectomy or transcervical resection of the endometrium (TCRE) for menstrual pain and bleeding. They examined with preoperative 2D and 3D ultrasound predominantly morphology of JZ (normal vs. serrated vs. adenomyosis of inner myometrium). They found that an ultrasonographic diagnosis of adenomyosis of the inner myometrium by 2D-TVS was not confirmed by histopathology in 19 of 42 (45%) women, and 17 (90%) of these had a serrated JZ. A 3D-TVS diagnosis of adenomyosis of the inner myometrium was not confirmed by histopathology in 11 of 33 (33%) women, and eight (73%) of these had a serrated JZ. Thus, most false positive cases had a serrated JZ. However, there were fewer women with a serrated JZ diagnosed as adenomyosis of the inner myometrium by 3D-TVS (n=8) than with 2D-TVS (n=17). Their findings regarding internal adenomyosis obviously cannot be extrapolated to disease expanding to deeper myometrium, and further studies are needed.

### Adenomyosis and subfertility

The relation of adenomyosis to infertility and subfertility also remains uncertain. This uncertainty partly relates to the fact that infertility is frequently multifactorial. Due to a large number of cofounders, large populations are needed in order to determine this association. On the other hand, the long- standing concept of adenomyosis being a disease of late reproductive and premenopausal years has recently been challenged and instead of hysterectomy, imaging techniques are currently used for its diagnosis in the majority of suspect cases (Bajot and Darai, 2018, Tellum et al., 2020). As a result, the recognition that adenomyosis may affect much younger populations led to investigation of its potential negative impact on female fertility.

Despite the theoretical impact of the presence of adenomyosis on female fertility, and its many molecular similarities with endometriosis, an established infertility factor, it is difficult to correlate the presence and clinical severity of this condition with the probability of spontaneous conception. Furthermore, adenomyosis frequently co-exists with endometriosis and other pathologies such as fibroids that can also have a negative impact on fertility. The possible underlying pathogenetic mechanisms of infertility in women with adenomyosis involve not only molecular changes of the eutopic endometrium that may affect implantation, (Benangiano et al., 2012; Benangiano et al., 2014b) but also abnormal peristaltic activity of the inner myometrium that may interfere with sperm transport (Kissler et al., 2007).

There is current ample evidence that the presence of adenomyosis is associated with the dysregulation of a large number of implantation-associated factors (HOXA10, LIF, MMP2, IL-6, cytochrome 450, and RCAS1), immune factors, pro-inflammatory mediators (IL-1β, CRH), markers of apoptosis and proliferation, and mediators of oxidative stress, leading to low uterine receptivity (Campo et al., 2012, Vannuccini et al., 2017). Additionally, adenomyosis in common with endometriosis is associated with the development of progesterone resistance (Campo et al., 2012; DeZiegler et al., 2010; Vannuccini et al., 2017). As a result of persistent local hyper-estrogenism dysregulated uterine peristalsis mediated by endometrial oxytocin and its receptors ensues, causing further trauma and endometrial invasion of the junctional zone (Garcia- Solares et al., 2018; Shaked et al., 2015). The altered eutopic endometrium displays a dysregulation of immune factors, markers of apoptosis or proliferation, inflammatory mediators, and oxidative stress resulting in low uterine receptivity (Campo et al., 2012).

Although many consider an increased thickness of JZ a sign of early adenomyosis, this has been disputed by Tocci et al. (2008) who believe that JZ disruption disease is a different pathological entity. The normal JZ itself when diffusely thickened and not irregular should be carefully distinguished from normal physiological thickness variability that occurs throughout the cycle in response to a varying hormonal environment (Brosens et al., 1995; Fusi et al.; 2006; Kishi et al., 2017). There is no doubt that establishing universally accepted imaging criteria for JZ thickness normality unrelated to early adenomyosis is crucial to avoid overdiagnosis of this condition and false clinical correlations.

A relatively good model for prospectively studying the effect of adenomyosis on conception and early pregnancy has been assisted reproduction. The potential detrimental effect of a thickened JZ at imaging on implantation and evolution of early pregnancy has been suggested by several authors. Unfortunately, many of these studies report on small numbers of patients. Chiang et al. (1999) suggested a link between miscarriage and uterine JZ dysfunction in infertile patients undergoing IVF and found that the spontaneous abortion rate was higher in women with a diffusely enlarged uterus on ultrasound imaging without distinct uterine masses compared with those with a normal uterus (66.7% vs. 21%, p=0.04). However, their clinical pregnancy rates were not statistically different (31.6% vs. 26.4%). Piver (2005) proposed that evaluation of JZ thickness with MRI is the best negative predictive factor of implantation failure, and an increase in JZ diameter is inversely correlated to implantation rate. Implantation failure was found to be high when the average JZ was greater than 7mm, possibly setting an upper limit of normality which is lower than the usual reported threshold for diagnosis of adenomyosis. Similarly, Maubon et al. (2010) in a prospective study of 152 infertile women who had a pelvic MRI prior to IVF, measured the average and maximum JZ thickness and correlated implantation outcomes both with JZ thickness and causes of infertility (endometriosis, tubal infertility, anovulation, male factor, and unexplained infertility) (48). The implantation failure rates in their series were 95.8% vs. 37.5% in the groups with a JZ > 7mm vs. < 7mm, respectively. Surprisingly, in this study the highest pregnancy rate (59.3%), was in the endometriosis group, known from other studies to be associated with the thickest JZ (Kunz et al., 2005).

In a recent metanalysis Younes and Tulandi (2015) examined the impact of adenomyosis on IVF outcome, including the effect on implantation. They found that patients with adenomyosis had significantly lower pregnancy (OR 0.70, 95%CI 0.60-0.90), and implantation (OR 0.66, 95%CI 0.49- 0.88) rates, compared with those without. They also observed that patients with diffuse adenomyosis have a tendency for lower pregnancy rates than those with focal disease (OR 1.36, 95%CI 0.67-2.75).

In another interesting study, Mavrelos et al. (2017) found that IVF patients with ultrasound findings of adenomyosis had significantly decreased clinical pregnancy rates, (29.2% vs. 42.6%, p=0.044, OR 0.68, 95%CI 0.47-1.00), and that the presence of ≥4 ultrasound features was a negative predictor for clinical pregnancy (OR 0.35, 95%CI 0.15-0.82), compared with those with no adenomyosis features. Their findings indicate that the more severe the disease, the higher is the possibility of decreased pregnancy rates. Unfortunately, in the majority of reported studies on the effect of adenomyosis on ART results, the imaging diagnostic criteria of this condition are vague and inconsistent.

A potentially important consequence of the accurate diagnosis of adenomyosis in infertile patients undergoing ART is the selection of an appropriate treatment protocol. Additionally, a universally accepted imaging classification of the severity and extent of adenomyosis would be of utmost importance to evaluate the prognosis of patients with this condition undergoing ART, assisting in the design of randomized studies evaluating different IVF protocols (Gordts et al., 2018). Park et al. (2016) reported on 214 IVF cycles in women with adenomyosis, comparing the IVF outcomes of fresh embryo transfer (ET) cycles with (N=147 – group A), or without (N=105 – group B) gonadotropin-releasing hormone (GnRH) agonist pre-treatment, and of frozen-thawed embryo transfer (FET) cycles following GnRH agonist treatment (N=43 – Group C). The clinical pregnancy rate in group C (39.5%) tended to be higher than those in groups B (30.5%) and A (25.2%) (Park et al., 2016).

### Adenomyosis and pregnancy-related complications

Epidemiologic studies have also shown that in women with adenomyosis the course of pregnancy may be complicated by several adverse events such as preterm labour with or without rupture of membranes (PPROM), placental abruption, pre-eclampsia and small for gestational age (SGA) (Buggio et al., 2018; Hashimoto et al., 2018). Delivery may be complicated by placental malpositions, postpartum haemorrhage, and caesarean hysterectomy (Vigano et al., 2015; Vlahos et al., 2017). It is unclear however, what the real impact of adenomyosis on pregnancy-related complications is, as in the majority of cases the diagnosis is made postnatally.

In a very recent metanalysis of 6 studies Razavi et al. (2019) reporting on 322 adenomyosis cases and 9420 controls attempted to shed light on the important question i.e. whether adenomyosis is associated with adverse pregnancy outcomes. In all included studies the diagnosis of adenomyosis was made with TVS, MRI, or a combination of the two imaging modalities. Despite having different objectives in terms of the adverse pregnancy outcome(-s) studied in relation to the presence or absence of adenomyosis, their observational nature, differences in selection of controls, and the potential effect of previous obstetric history and other risk factors on pregnancy complications that were not eliminated through multivariate analysis, this metanalysis produced interesting conclusions: women with adenomyosis had an increased likelihood of preterm birth (OR, 3.05; 95%CI, 2.08- 4.47; p<0.001), SGA (OR, 3.22; 95%CI, 1.71-6.08; p<0.001), and pre-eclampsia (OR, 4.35; 95%CI, 1.07-17.72; p=0.042). However, there was no evidence of an association between adenomyosis and foetal malpresentation. Similarly, Bruun et al. (2018) in a large metanalysis including studies on pregnant patients with endometriosis and/or adenomyosis, found that those with adenomyosis had an increased risk of both preterm delivery (OR of 3.09 (95% CI; 1.88-5.09)) and SGA (OR: 3.23, 95% CI; 1.71-6.09). Studies on adenomyosis were much less in number compared with those reporting on endometriosis, therefore firm conclusions could not be drawn from this metanalysis other than to suggest close monitoring of these patients during pregnancy.

The pathogenetic mechanism underlying these adenomyosis-related pregnancy complications probably involves several different aspects. Preterm labour with or without PPROM may be caused by an activated systematic or uterine inflammatory process or infection. Levels of prostaglandins and cytokines in the peritoneal fluid are higher among women with adenomyosis than among controls (Juang et al., 2007). Local and systematic inflammation triggers myometrial vasoconstriction and stimulates cervical ripening (Vannuccini et al., 2016). Additionally, an implantation and a placentation defect commonly underlies pre-eclampsia, preterm delivery and foetal growth restriction. In the case of adenomyosis, pronounced changes of the endometrium- myometrium interface possibly interferes with normal placentation through impaired spiral artery remodelling (Brosens et al., 2010; Brosens et al., 2013). Furthermore, it has been suggested that an additional cause of SGA in cases with adenomyotic uteri may be the increased blood flow shift towards the adenomyotic lesion rather than the placenta (Yorifuji et al., 2013).

Unfortunately, no prospective comparative study exits correlating the depth and extent of adenomyosis with the probability of developing pregnancy complications. Such a study should obviously rely on imaging diagnosis and taking into account what has been discussed above should necessarily include cases with adenomyosis of the inner myometrium that have an increased potential to develop impaired placentation. As with infertility and other clinical correlations, the true effect of adenomyosis on pregnancy-related complications relies largely upon a universally accepted imaging diagnosis, preferably offering classification of its extent and severity.

### Adenomyosis and pelvic co-morbidities

Several studies on patients undergoing hysterectomy for either chronic pelvic pain or abnormal uterine bleeding have shown that adenomyosis may co-exist with a variety of uterine and non-uterine conditions that may be responsible for the same spectrum of symptoms. These include endometriosis, leiomyomas, endometrial polyps, and less commonly endometrial hyperplasia, and uterine malignancies. In the study of Li et al. (2014) reporting on 710 hysterectomy cases found with adenomyosis, 343(48.3%) had adenomyosis alone, 158(22.3%) adenomyosis and endometriosis, 129(18.2%), adenomyosis and fibroids, and 80(11.3%) all three conditions combined. It appears that hyper- oestrogenism is the common denominator of all these conditions (Bergeron et al., 2006; Vercellini et al., 2014; Reis et al., 2016).

The prevalence of adenomyosis in symptomatic cases with histologically proven endometriosis, has been reported to be 40% in a recent study (Lazzeri et al., 2014). Naftalin et al. (2012) have reported that 48.7% of patients with deep infiltrative endometriosis, are also diagnosed with adenomyosis. Endometriosis has a spectrum of symptoms similar to that of adenomyosis including chronic pelvic pain and abnormal uterine bleeding. It is also a significant factor of female subfertility. Co-existence of endometriosis and adenomyosis in the same patient is always a source of controversy regarding the attribution of specific symptoms to each condition. Although they share several pathogenetic and clinical characteristics they also have considerable differences, for example, in terms of molecular characteristics of the eutopic endometrium, such as the leukocyte population and apoptosis markers. There is also some evidence of differences in cytokines and inflammatory mediators (Benangiano et al., 2014).

There is recent evidence that adenomyosis may develop earlier in life in women with endometriosis (Kunz et al., 2007; Chapron et al., 2017). Kunz et al. (2007) performed MRI on 227 women with and without endometriosis who were stratified into 4 age groups (17-24, 25-29, 30-34, and >35 years). They demonstrated that increasing thickness of the dorsal JZ (an equivalent of adenomyosis according to their definition), commenced early in the third decade of life, and progressed steadily during the fourth decade in patients with endometriosis. On the contrary, women without endometriosis showed almost no sign of adenomyosis up to the age of 34 years (average JZ thickness >11 vs. <8mm). After 34 years both groups demonstrated a marked and in parallel increase in the thickness of JZ representing adenomyosis, a finding which probably indicates an age-related pathophysiological continuum of this condition.

Another parameter that further complicates this issue is the lack of reliability of the criteria used to make the diagnosis of adenomyosis, especially with imaging methods when other uterine pathologies and in particular fibroids are present in the same patient. The diagnosis of adenomyosis in such cases can in theory be strengthened by presence of disease-specific menstrual symptoms (D-SMS). Taran et al. (2012) reporting on 291 hysterectomy cases treated for adenomyosis and/ or fibroids found a significantly higher incidence of D-SMS in patients with adenomyosis alone (p=0.008). However, no significant differences were observed for the occurrence of hypermenorrhoea, menorrhagia/metrorrhagia, dysmenorrhea, pain, or dyspareunia, between the three groups of patients. Their results strengthen the doubt of what really constitutes D-SMS in adenomyosis. On the other hand, technical issues may also complicate the diagnosis. In the study of Naftalin et al. (2012) of 20 cases who underwent hysterectomy within 2 years from imaging diagnosis of adenomyosis, 4 (20%) patients with multiple fibroids were excluded from comparison between ultrasound and histology diagnosis, due to the difficulty to obtain systematic representative sections from every part of the specimen to study adenomyosis.

The same investigators, in their group of 157 cases with adenomyosis reported a prevalence of 27.4% of intramural/subserous fibroids, 6.4% submucous fibroids, and 1.9% endometrial polyps. Their multivariate analysis for subjective assessment showed that all three pathologies were significantly associated with menorrhagia, but not dysmenorrhea (Naftalin et al., 2014; Naftalin et al., 2016). On the contrary, Li et al. (2014) in their group of 710 adenomyosis cases using a logistic regression model demonstrated that the presence of fibroids was not associated positively with either complaint, whereas presence of endometriosis in their series was positively associated with dysmenorrhea and chronic pelvic pain, and negatively with menorrhagia.

It is therefore possible that in several women with other uterine and pelvic diseases that are not subjected to hysterectomy, the diagnosis of adenomyosis will be missed, and symptoms caused by it will be attributed to other causes that are easier to identify with non-invasive tools. Additionally, differences in study populations (age, presence and severity of symptoms), method of final diagnosis (imaging, histology), and design of studies (prospective, retrospective), may well be responsible for discrepancies in the results of clinical correlations in patients diagnosed with adenomyosis.

### Symptomatology and the wider spectrum of adenomyosis

What has been discussed above refers predominantly to diffuse adenomyosis defined as the extensive form of the disease, characterized by foci of endometrial mucosa (glands and stroma) scattered throughout the uterine musculature (Grimbizis et al., 2014). Grimbizis G, et al., proposed a new classification in an attempt to include all common and uncommon forms of this disease under this heading (Grimbizis et al., 2014). Focal forms of adenomyosis such as adenomyomas including the less common cystic variables (adult and juvenile adenomyomas) possibly have a non-TIAR pathogenetic mechanism of development and exhibit distinct imaging and clinical profiles (Gordts et al., 2018).

Focal adenomyomas of the nodular type most frequently develop in patients in their late 30s (Gilks et al., 2000; Grimbizis et al., 2008). Their size may vary considerably, and it has been reported to range from 0.3-17cm in a series of 30 cases treated with hysterectomy (Gilks et al., 2000). They commonly present with worsening dysmenorrhea that may be accompanied by menorrhagia or meno-metrorrhagia. Occasionally, they may be diagnosed on the occasion of a pregnancy complication (Grimbizis et al., 2008). Their appearance on both TVS and MRI is similar to that of fibroids and especially those exhibiting cystic degenerative changes, and although experienced groups on both imaging modalities have reported on specific characteristics that facilitate the differential diagnosis (Exacoustos et al., 2014; Song et al., 2011), it is frequently made during fertility-sparing surgery. The adult cystic variety is a rare form of focal adenomyosis, and few of these cases may present as giant cystic tumours arising from the uterus from a narrow pedicle. Several cases with this type of adult cystic adenomyomas are completely asymptomatic and are misdiagnosed as adnexal cysts. On the contrary, the juvenile type commonly presents with debilitating dysmenorrhea dating as early as menarche requiring prompt management. The majority of women with adult cystic adenomyomas are also significantly younger compared with those bearing diffuse adenomyosis (Protopapas et al., 2008).

Brosenset al. (2015) analyzed all cases of cystic adenomyosis that had been reported until 2012. The most striking characteristic in the majority of these patients was indeed their significantly younger age at diagnosis. The majority had an early onset of symptoms, predominantly severe dysmenorrhea that dated since the patients’ onset of menstruation. Menorrhagia and irregular uterine bleeding were by far less common.

Polypoid adenomyomas on the other hand represent an even rarer form of focal lesion that also develops more commonly in younger patients. They invariably protrude into the uterine cavity or the endocervical canal and present with abnormal uterine bleeding. They are frequently misdiagnosed as endometrial polyps and are treated as such (Mikos et al., 2019, Protopapas et al., 2016). Occasionally, they may co-exist with other forms of adenomyosis that will complicate the clinical picture causing chronic pelvic pain symptoms (Protopapas et al., 2017). They may also present with histological atypia or co-exist with endometrial hyperplasia and adenocarcinoma (Grimbizis et al., 2017; Protopapas et al., 2016).

### Conservative surgery and symptomatic relief

In symptomatic patients that are subjected to conservative surgery for adenomyosis it is easier to make clinical correlations. The effect of surgery on symptoms relief, at least in theory, is a clear indicator of the morbidity caused preoperatively by the disease, especially in patients without co-existing pathologies. Additionally, a histological diagnosis of adenomyosis will be made despite weaknesses and lack of universally accepted criteria.

In a recent metanalysis, Mikos et al. (2020) analysed the results of 19 studies and a total of 1843 patients submitted to fertility-sparing surgery for adenomyosis. They have shown that complete resection of the disease was related with improvements in pain and menorrhagia, and a reduction in uterine volume by a factor of 6.2, 3.9, and 2.3, respectively. Regarding the same outcomes, partial excision was related with improvements of symptoms and size reduction by a factor of 5.9, 3.0, and 2.9, respectively. In studies with mixed volume of patients, (complete and partial excision) the corresponding factor-figures were 4.0, 6.3, and 5.1, respectively.

It also appears that patients with focal adenomyosis have the best chances of symptom improvement or resolution after fertility-sparing surgery. Percentages of pain reduction has been reported to range from 45-80% with reductions in dysmenorrhea reported as high as 98%. Percentage reductions in uterine bleeding are in general less pronounced and range from 59-75%. Patients with diffuse and extensive disease have a significant variability of pain reduction (18-91%), with an average of 60% (46% for dysmenorrhea). A reduction of uterine bleeding after surgery in such cases has been reported to reach an average of 60% (48-71%). This variability in the later cases probably reflects different operative techniques and the extent of surgery applied in cases with diffuse adenomyosis (Berlanda et al., 2016).

In another recent systematic review of 18 studies and 1396 infertile patients Tan et al. (2018) analysed the effects of surgical treatment of adenomyosis on reproductive outcomes. They demonstrated that overall, the reproductive outcome was better in cases with focal adenomyosis compared to those with diffuse disease, in particular total pregnancy rates (52.7 vs 34.1%), and successful delivery rates (43.5 vs. 25.0%). On the contrary, miscarriage rates were similar in both groups (21.1 vs. 21.7%). No significant differences were observed between groups regarding natural conception and ART with or without GnRH agonist pre-treatment.

It is therefore evident, that adenomyosis may indeed be responsible for all the afore-mentioned types of symptoms and signs that can be alleviated to a varying extent with surgery. There is also a chance of improvement in the reproductive outcomes which is less for diffuse disease (Kunz et al., 2005; Mikos et al., 2020). Occasionally, this will come at a price, i.e. the rare risk of uterine rupture during pregnancy due to a defective scar, which has been reported to be of the order of 6.8% in cases with diffuse disease (0% in focal) (Tan et al., 2018). There is no doubt that a solid system of preoperative classification of the extent and severity of adenomyosis correlating it with symptoms and potential pregnancy complications, would considerably assist decision- making during conservative surgery in order to avoid unnecessary radicality.

### Can existing pathogenetic theories explain discrepancies in clinical profiles?

There is no doubt that we still have a long way to go regarding the clarification of the natural history of adenomyosis. The reasons rest mainly on the inability to make a firm non-invasive diagnosis from early reproductive life and the huge difficulties in properly following up a large asymptomatic cohort of young women to their menopause. Nevertheless, we believe that a correlation between the proposed pathogenetic theories and the clinical spectrum of adenomyosis can be attempted based on existing studies.

Present data indicate that adenomyosis may indeed appear as an asymptomatic entity in genetically and epigenetically predisposed females. Those women developing symptoms from menarche possibly bear lesions that rest in ectopic locations since their embryonic life. The appearance of symptoms during adolescence and early reproductive life may indicate both a congenital aetiology and an epigenetic mechanism of early change of lesions that possesses a progressive character leading to the gradual deterioration of menstrual pain in particular. Menarche will obviously result in onset of dysmenorrhea in intra-myometrial isolated non-communicating with the endometrial cavity ectopic lesions bearing functional endometrium. Early disease involving the JZ will present with increased menstrual loss that may not have a rapid progressive nature due to the repair mechanism that will temporarily isolate minor lesions from eutopic endometrium, and possibly prevent cross-talk. A thickened JZ may be considered an early stage of the TIAR mechanism when no permanent changes of the inner myometrium have occurred. Whether it will evolve to typical adenomyotic lesions, will depend on the longevity of the insult, subsequent molecular and genetic changes, and the operation of risk factors such as pregnancy and uterine surgery. Therefore, recognition of JZ thickening may be considered an indicator of an increased risk for developing adenomyosis in later life.

Dysmenorrhea that has a more constant relation with disease severity and extent, will be gradually aggravated by further changes of lesions resting in deeper myometrium – a more distant and foreign to the lesion’s environment. Development of deeper lesions in the mid-reproductive years when dysmenorrhea usually appears first, may be associated with both a TIAR mechanism and/or metaplasia of progenitor stem cells. The frequent co-existence of endometriosis in this age group commonly causing worsening dysmenorrhea and sharing similar pathogenetic pathways with adenomyosis may be an important contributor to pain symptoms. Menorrhagia may re-appear in late reproductive and premenopausal years in relation to the evolution of adenomyosis severity - by involving more myometrium and increasing its vascularity - and as a result of the operation of risk factors associated with reproduction, including reproductive and obstetric surgery, and uterine aging, that will inflict further epigenetic changes to adenomyotic lesions, or re-activate and deteriorate a dormant TIAR mechanism. Co-existing uterine pathologies of mid-40s and beyond, may also contribute to menorrhagia. Asymptomatic lesions on the other hand, that may be recognized up to pre-menopause may have never been exposed to an adequately toxic environment and molecular insults to turn them into a disease process.

## Conclusion

As a result of recent imaging studies, including both transvaginal sonography and MRI, there is currently sufficient evidence indicating that adenomyosis, in contrast to previous beliefs that it mainly affects multiparous women of the late reproductive years, may appear early in life even in nulliparous women without classic risk factors. Adenomyotic lesions may be found in variable depths from the endometrium-myometrium interface, indicating different pathogenetic mechanisms between diffuse (internal and external), and focal adenomyosis. Severity of adenomyosis is commonly age-related, with various risk factors playing a role in its evolution. Co-existing endometriosis accelerates JZ thickening which possibly indicates early internal adenomyosis, whereas infiltrative disease is commonly found together with severe external adenomyosis. Progressive JZ thickening occurs from mid-30s - though to a lesser extent - also in unaffected women indicating that adenomyosis may also be a process related to uterine aging. Despite the fact, that the classic complex of adenomyosis-related symptoms and their timing during its evolution has recently been challenged, the majority of studies indicate that severe dysmenorrhea remains the most reliable indicator of its severity and extent.

Although significant work has been done so far by many experts in both fields, an urgent need to further clarify the criteria for both imaging and histological diagnosis of adenomyosis and develop a universally accepted classification of its spectrum, extent, and severity still exists. This should also take into account clinical correlations relating adenomyosis to severity of clinical symptoms such as pain and abnormal uterine bleeding, and its potential negative effect on fertility. Well-designed prospective studies are urgently needed to clarify the potential evolution of adenomyosis from an asymptomatic state to a disease.
